# Plasma pro-atrial natriuretic peptide to estimate fluid balance during open and robot-assisted esophagectomy: a prospective observational study

**DOI:** 10.1186/s12871-017-0314-6

**Published:** 2017-02-03

**Authors:** Rune Broni Strandby, Rikard Ambrus, Niels H. Secher, Jens Peter Goetze, Michael Patrick Achiam, Lars Bo Svendsen

**Affiliations:** 1 Department of Surgical Gastroenterology, University of Copenhagen, Rigshospitalet, Blegdamsvej 9, DK-2100 Copenhagen-Ø, Denmark; 2 Department of Anesthesiology, University of Copenhagen, Rigshospitalet, Blegdamsvej 9, Copenhagen-Ø, DK-2100 Denmark; 3 Department of Clinical Biochemistry, University of Copenhagen, Rigshospitalet, Blegdamsvej 9, Copenhagen-Ø, DK-2100 Denmark

**Keywords:** Central blood volume, Abdominal surgery, Fluid balance, Plasma-atrial natriuretic peptide

## Abstract

**Background:**

It remains debated how much fluid should be administered during surgery. The atrial natriuretic peptide precursor proANP is released by atrial distension and deviations in plasma proANP are reported associated with perioperative fluid balance. We hypothesized that plasma proANP would decrease when the central blood volume is compromised during the abdominal part of robot-assisted hybrid (RE) esophagectomy and that a positive fluid balance would be required to maintain plasma proANP.

**Methods:**

Patients undergoing RE (*n* = 25) or open (OE; *n* = 25) esophagectomy for gastroesophageal cancer were included consecutively in this prospective observational study. Plasma proANP was determined repetitively during esophagectomy to allow for distinction between the abdominal and thoracic part of the procedure. The RE group was 15° head up tilted during the abdominal procedure.

**Results:**

The blood loss was 250 (150–375) (RE) and 600 ml (390–855) (OE) (*p* = 0.01), but the two groups of patients were provided with a similar positive fluid balance: 1705 (1390–1983) vs. 1528 ml (1316–1834) (*p* = 0.4). However, plasma proANP decreased by 21% (*p* < 0.01) during the abdominal part of RE carried out during moderate head-up tilt, but only by 11% (*p* = 0.01) during OE where the patients were supine. Plasma proANP and fluid balance were correlated in the RE-group (*r* = 0.5 (0.073–0.840), *p* = 0.02) and tended to correlate in the OE group (*r* = 0.4 (−0.045–0.833), *p* = 0.08).

**Conclusion:**

The results support that plasma proANP decreases when the central blood volume is compromised and suggest that an about 2200 ml surplus administration of crystalloid is required to maintain plasma proANP during esophagectomy.

**Trial registration:**

Clinicaltrials.gov (NCT02077673). Registered retrospectively February 12^th^ 2014.

## Background

Fluid administration affects outcome after surgery [1–3], but it remains debated how much fluid should be administered and how the volume load is to be evaluated [4, 5]. For colorectal surgery a “restricted” fluid regimen seems profitable in regard to cardiopulmonary complications and tissue healing [3, 6]. On the other hand, patients going through laparoscopic cholecystectomy appear to benefit from a “liberal” fluid regimen [1], probably because the patients are head-up tilted. Deviations in postoperative outcome relate likely to how well the central blood volume (CBV) is maintained during surgery. Consequently, so-called individual goal-directed fluid therapy aims at maintaining a CBV that does not limit, e.g. stroke volume (SV) during surgery, eventually based on a report of SV by minimally invasive apparatus [5, 7].

We considered that plasma atrial natriuretic peptide (ANP) would indicate whether filling of the heart is maintained during surgery. ANP – but not “brain” natriuretic peptide [8] - reacts rapidly to a reduction in CBV, e.g. during head-up tilt [9] or sitting or standing up [8] as with pressure breathing [10] demonstrating independence of even a large increase in central venous pressure [11]. Compared to plasma ANP, plasma pro-ANP (proANP) is stable with a half-life of 60–120 min [12] and during cystectomy, plasma proANP decreases with the perioperative blood loss and, conversely increases with a positive fluid balance when administration is based mainly on lactated Ringer’s solution (LR) [13].

We determined plasma proANP and perioperative fluid balance during open (OE) and robot-assisted hybrid (RE) esophagectomy. During esophagectomy CBV could be compromised not only by an eventual blood loss but also by, e.g. epidural analgesia during OE and head-up tilt and abdominal CO_2_ insufflation during RE. We hypothesized that plasma proANP would decrease when CBV is compromised during the abdominal part of RE and that a positive fluid balance would be required to maintain plasma proANP when fluid administration is based mainly on LR.

## Methods

This prospective non-randomized study was a secondary data analysis of a clinical trial directed to monitor gastric microcirculation during RE and OE esophagectomy (ClinicalTrials.gov, ID: NCT02077673) [14] as approved by the Scientific Ethical Committees, Capital Region, Denmark (H-2-2013-101). Patients were consecutively included between December 2013 and April 2015. Oral and written informed consent was provided at least 1 day before surgery. All patients with biopsy verified adenocarcinoma of the gastroesophageal junction eligible for a two-stage procedure with an abdominal and a thoracic part (Ivor Lewis esophagectomy) [15] were candidates for the study (Table 1). Data were collected by the investigators and remained confidential throughout the trial. Patients were excluded from the study if consent was withdrawn or disseminated disease was evident, i.e. only patients for whom the operation was completed were included.

**Table 1:** Patient characteristics for patients undergoing robot-assisted hybrid (RE) or open esophagectomy (OE)

	RE (*n* = 25)	OE (*n* = 25)	*P*-value
Age, years	64.8 (±10.4)	68 (±7.9)	0.1
Male sex, *n* (%)	22 (88.0)	20 (80.0)	0.5
BMI, kg/m^2^	25.2 (±3.3)	25.8 (±5.1)	0.15
Alcohol, earlier abuse, *n* (%)	3 (12.0)	2 (8.0)	1.0
Tobacco, current & former, *n* (%)	22 (88.0)	22 (88.0)	1.0
ASA-classification ≥3, *n* (%)	5 (20.0)	12 (48)	0.04
Hypercholesterolemia, *n* (%)	1 (4.0)	7 (28.0)	0.03
Hypertension, *n* (%)	10 (40.0)	12 (48.0)	0.6
Diabetes, *n* (%)	5 (20.0)	8 (32.0)	0.4
Heart disease, *n* (%)	1 (4.0)	3 (12.0)	0.7
Pulmonary disease, *n* (%)	1 (4.0)	5 (20.0)	0.1
Duration of procedure, minutes	254 (±34.0)	239 (±41.0)	0.9
LOS, days	13 (±7.0)	15 (±8.0)	0.3

*BMI* body mass index, *ASA* American Society of Anesthesiologists classification, *LOS* length of hospital stay. Heart disease: ischemic heart disease, arrhythmias, and valve insufficiency. *P*-values by univariate analyses. Values are mean with standard deviation (SD) unless stated otherwise

### Anesthesia and interventions

An i.v. line was established followed by a thoracic epidural catheter (Th7-Th9) and its position was evaluated with the response to administration of 3 ml 2% lidocaine with adrenaline (SAD, Amgros I/S, Denmark). Induction of anesthesia was with propofol (2.0 mg/kg) and remifentanil (0.5 μg/kg) followed by placement of a double-lumen endobronchial tube after neuromuscular blockade by cisatracurium. Anesthesia was maintained by propofol (5–10 mg/kg/h) and remifentanil (1.75–2.25 mg/h) and ventilation was adjusted to an end-tidal CO_2_ tension of 28–32 mmHg (Dräger CATO; M32040, Lübeck, Germany). Guided by ultrasound a central venous catheter was established via the right jugular vein for infusion of fluids and, if considered necessary vasopressors (Table 2). LR (3 ml/kg/h) was supplemented by 5% Voluven® or human albumin 5% if considered in need by the anesthesiologist. Red blood cells were administered when hemoglobin was lower than 4.5 or 5.5 mmol/l if the patient was known with cardio-pulmonary disease. For epidural anesthesia 4 ml bupivacaine (5 mg/ml, SAD) was administered before start of the procedure in both groups. Analgesia was maintained with bupivacaine (4 ml/h) with morphine (comb. 2.5 mg and 50 microgram/ml, SAD, Amgros I/S, Denmark) before start of laparotomy in the OE-group and before the thoracotomy in the RE-group.

**Table 2:** Perioperative fluid administration during open (OE) and robot-assisted esophagectomy (RE)

	OE (*n* = 25)	RE (*n* = 25)	*P*-value
Fluid administration, ml^a^	2600 (2400–3166)	2500 (2150–2825)	0.2
Electrolytes, ml^b^	1993 (1725–2475)	2000 (1700–2300)	0.7
Human albumin, Voluven & PRBC, ml	500 (500–938)	500 (250–750)	0.2
Ephedrine, mg	2.5 (0–14)	5.0 (0–13)	0.9
Phenylephrine, mg	0.1 (0–0.3)	0.2 (0–0.4)	0.9
Vasopressor infusion, ml/min	0.14 (0.1–0.2)	0.12 (0.1–0.2)	0.6
Fluid loss, ml^c^	1018 (839–1345)	655 (445–1065)	0.01
Blood loss, ml	600 (390–855)	250 (150–375)	0.01
Diuresis, ml	410 (296–599)	345 (300–490)	0.5
Fluid balance, ml^d^	1528 (1316–1834)	1705 (1390–1983)	0.4

Values are medians with interquartile range. *P-*values by Mann–Whitney U-test. ^a^Fluid administered during anesthesia including medicine and packed red blood cells (PRBC), ^b^primarily lactated Ringer’s solution, ^c^Fluid loss = diuresis and blood loss, ^d^Fluid balance = fluid infusion – fluid loss. For vasopressor infusion norepinephrine or phenylephrine were used

The OE patients were supine during the abdominal part of the procedure while RE patients were tilted 15° head-up for use of a da Vinci System (5.0 robotic, Intuitive Surgical Inc., Sunnyvale, CA, USA). The thoracotomy was on the right side in left-lateral decubitus position.

Heart rate (HR), SV, mean arterial pressure (MAP), cardiac output (CO), and systemic vascular resistance (SVR) were monitored by modified Model flow technology (Nexfin®, BMEYE B.V., Amsterdam, The Netherlands) through a radial artery catheter in the non-dominant arm [16]. Markers in the Nexfin file were: A after induction of anesthesia (baseline); B following laparotomy (OE) or pneumoperitoneum (RE); C 15 min after start of the procedure; D after mobilization of the stomach, and E following abdominal closure (OE) or CO_2_ desufflation (RE); F following the gastric remnant pull to the thorax; G formation of the anastomosis, and H following closure of the thorax. Also, arterial blood samples were obtained at each of these events for determination of plasma proANP. The samples were centrifuged at 3000 rpm for 10 min at 4 °C and plasma stored at −80 °C until analysis.

Fluid balance was calculated following closure of the thorax: LR, human albumin 5%, Voluven®, packed red blood cells, and medicine vs. the blood loss and diuresis. Also, a separate balance for colloid and crystalloids was estimated.

### Plasma proANP

Plasma proANP was measured with an automated method from Thermo-Fisher (the Kryptor Plus platform) that directs antibodies against epitopes within the mid-region of the precursor [17] and is validated with excellent performance in non-heart failure patients against a gold standard immunoassay [18–20].

### Statistics

Statistics was carried out by IBM SPSS® version 22.0.0 (SPSS, Inc., IL, USA) and graphs constructed (Graph Pad Software Inc., CA, USA). Baseline characteristics were evaluated with chi-square or Fisher’s exact test for nominal variables and *t*-test and Mann–Whitney U-test for continuous variables depending on whether data were normally distributed. To assess changes in plasma proANP vs. fluid balance linear regression analysis was used. Changes in hemodynamic variables and in plasma proANP during surgery were analyzed using the Friedman’s test. Post hoc comparisons with Bonferroni correction were applied if the initial test was significant. Values are expressed as mean with standard deviation or medians with interquartile range (IR) and a *p*-value ≤0.05 was considered statistical significant.

## Results

For RE 25 patients were enrolled consecutively and there were 26 patients in the OE-group, but one OE patient was excluded because the procedure was changed to total gastrectomy. Patients in the OE group demonstrated a higher ASA-score ≥ 3 (*p* = 0.04) and a higher prevalence of hypercholesterolemia (*p* = 0.03) (Table 1).

### Fluid balance and hemodynamics

The intraoperative blood loss was 250 ml (150–375 IR) in the RE-group and 600 ml (390–855) during OE (*p* = 0.01), while fluid administration was similar (2500 (2150–2825) and 2600 ml (2400–3166), respectively) (*p* = 0.20, Table 2). Thus, the fluid balance was positive by 1705 (1390–1983) (RE) and 1528 ml (1316–1834) (OE), *p* = 0.40.

**Fig. 1:**
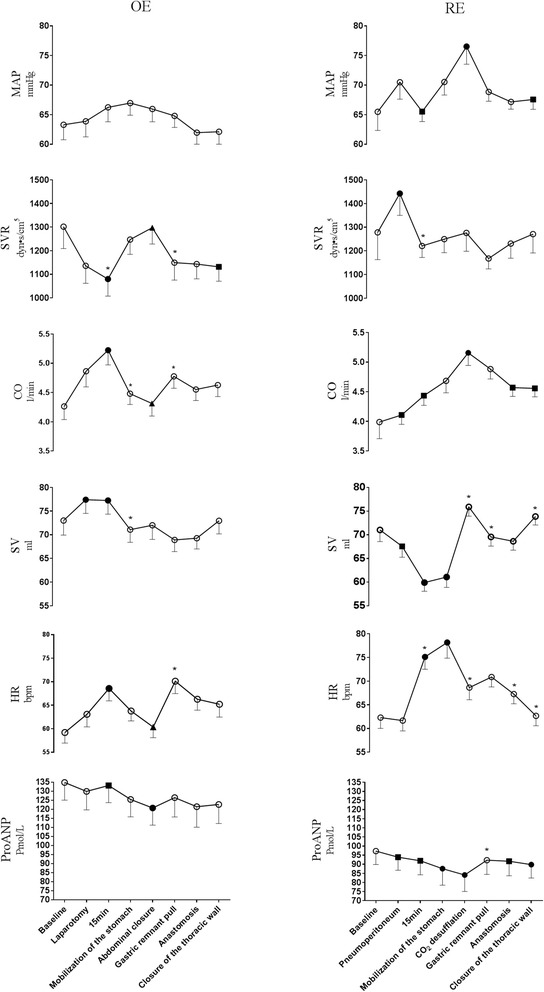
Hemodynamic variables and plasma proANP during esophagectomy. Values are mean +/− SEM. ○ No change. *Different from previous value, *p* < *0.05.* ● Different from ‘baseline’, *p* < *0.05.* ■ Different from ‘CO2 desufflation/abdominal closure’, *p* < *0.05.* ▲ Different from ’15 min’, *p* < *0.05*

Hemodynamic variables during the procedures are presented in Fig. 1. In the RE-group CO (*p* = 0.02) and HR (*p* < 0.01) increased from induction of pneumoperitoneum (B) to mobilization of the stomach (D). Meanwhile SV decreased (*p* < 0.01) and SVR increased after pneumoperitoneum was established (*p* = 0.04) but returned to baseline 15 min after start of the procedure (C) (*p* = 0.04) at a stable MAP (*p* = 1.00). Following CO_2_ desufflation (E) and termination of head-up tilt, MAP (*p* = 0.02), SV (*p* < 0.01), and CO (*p* < 0.01) increased while HR decreased (*p* = 0.01) leaving SVR stable (*p* = 1.00). During the thoracic part of the procedure MAP (*p* = 0.05), CO (*p* < 0.01), SV (*p* = 0.04), and HR (*p* = 0.02) decreased towards baseline levels, while SVR was stable (*p* = 0.31). However, SV (*p* = 0.01) increased following closure of the thorax.

During OE, HR (*p* < 0.01), SV (*p* = 0.05) and thus CO (*p* < 0.01) increased from baseline (A) to 15 min after start of the procedure (C). MAP remained stable (*p* = 0.70) and hence SVR decreased (*p* = 0.02). By abdominal closure HR (*p* = 0.02), CO (*p* < 0.01), and SV (*p* = 0.18) decreased towards baseline levels. MAP (*p* = 0.73) was stable and therefore SVR increased (*p* = 0.01). During the thoracic part of the procedure SVR (*p* = 0.01) decreased by gastric remnant pull with a concomitant increase in HR (*p* < 0.01) and CO (*p* < 0.01). Hemodynamic variables remained at these levels until closure of the thorax. There was no significant difference in vasopressor administration between the two groups of patients (Table 2).

### Plasma proANP

**Fig. 2:**
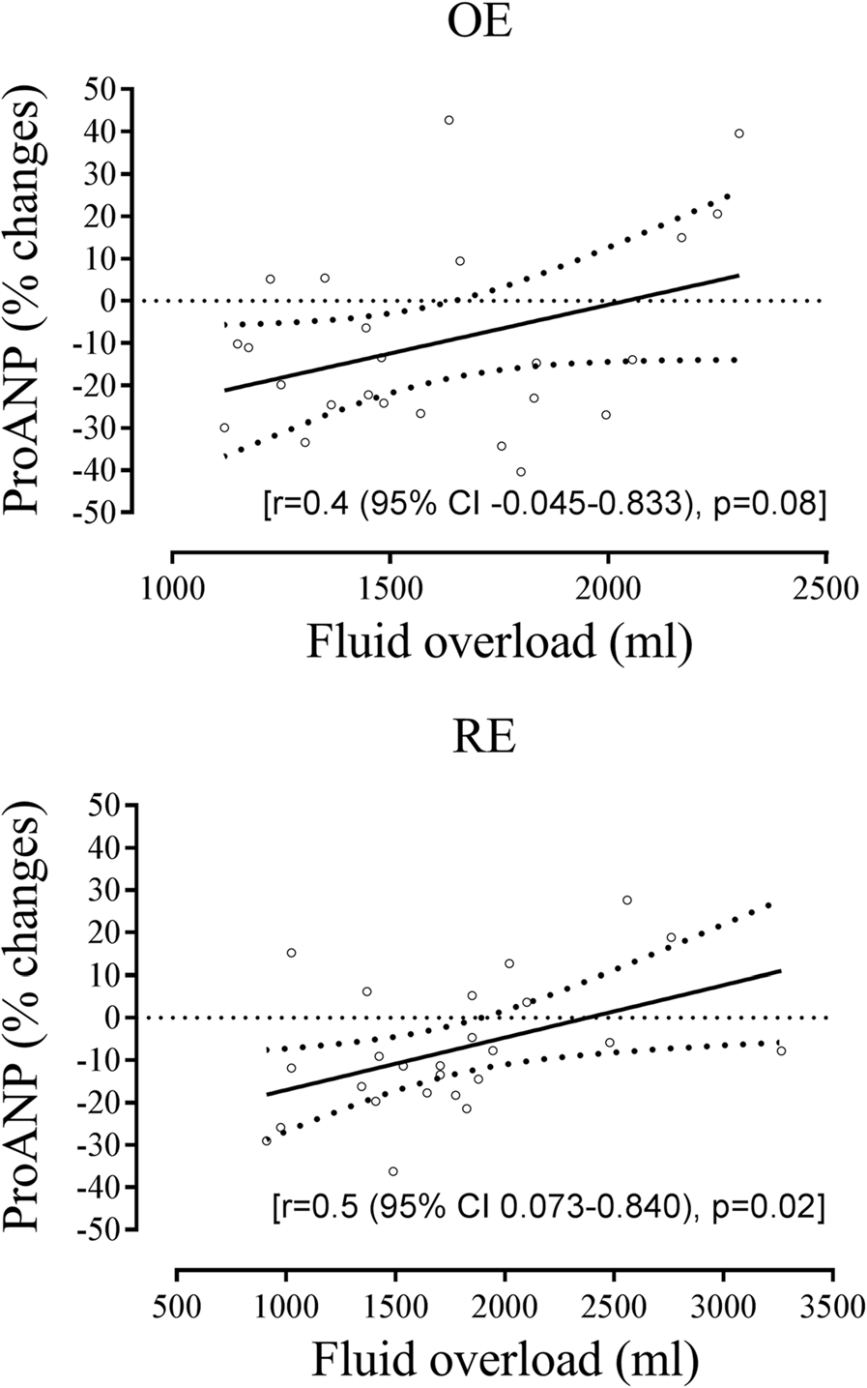
Plasma ProANP in relation to fluid balance during open (OE) and robot assisted esophagectomy (RE). Change in plasma proANP from start (baseline) to end of surgery (closure of the thorax). Regression line with 95% CI. *Horisontal broken line* indicates no change in proANP

In the OE-group plasma proANP decreased by 11% (*p* = 0.01) during the abdominal part of the procedure, but by 21% in the RE-group (*p* < 0.01). During the thoracic part of the procedure plasma proANP remained stable in the OE-group (*p* = 1.00), but increased in the RE-group from CO_2_ desufflation (E) to gastric remnant pull (F) (*p* = 0.01) (Table 3 and Fig. 1). However, plasma proANP was lower at thoracic closure (H) than at baseline (A) in the RE-group (*p* = 0.01). Linear regression between plasma proANP and fluid balance showed an *r*-value of 0.4 ((−0.045–0.833), *p* = 0.08) in the OE-group and 0.5 ((0.073–0.840), *p* = 0.02) for the RE patients (Fig. 2).

**Table 3:** Plasma proANP during robot-assisted (RE) and open esophagectomy (OE)

	RE (pmol/L, *n* = 25)	OE (pmol/L, *n* = 25)
Baseline	95 (64–125)	135 (102–161)
Pneumoperitoneum/Laparotomy	91 (60–119)	118 (94–168)
15 min after start of procedure	83 (59–114)	133 (96–173)
Mobilization of the stomach	81 (55–102)	123 (95–158)
CO_2_ desufflation/abdominal closure	74 (53–99)^a^	120 (82–157)^b^
Gastric remnant pull	89 (58–117)^c^	115 (88–158)
Anastomosis	87 (57–122)	101 (78–157)
Closure of the thorax	85 (56–119)^b^	104 (87–155)

Values are medians with interquartile range. *P-*values by Friedman’s test

^a^ *p* < 0.01 different from baseline within the group

^b^ *p* = 0.01 different from baseline within the group

^c^ *p* = 0.01 different from ‘CO_2_ desufflation’ within the group

## Discussion

Plasma proANP was determined repetitively during surgery to allow for a distinction between the abdominal and thoracic part of Ivor Lewis esophagectomy for which CBV could be compromised by the head-up tilt used for the abdominal part of the robot-assisted procedure [11, 21]. In fact, plasma proANP decreased twice as much during the abdominal part of RE (by 21%) than during OE (by 11%) despite fluid balance was positive by about 1700 ml (by the end of surgery) in both groups and the blood loss largest during OE (600 vs. 250 ml). Furthermore, plasma proANP, MAP, and CO increased while HR decreased after CO_2_ desufflation and termination of head-up tilt. Together, these observations support that CBV is reduced during surgery and especially so if there is a restrain on venous return to the heart by head-up tilt and abdominal CO_2_ insufflation.

The ratio between the interstitial fluid space and plasma is about 1:5 and it is assumed that only 20–25% of the administered LR remains in plasma [22, 23] and a separate calculation for the administered crystalloid and colloid was conducted (Table 2). If 25% of the administered 2000 ml of LR is taken to remain within the vessels together with 5% albumin/voluven® and red blood cells, the intravascular fluid balance would be +350 ml in the RE-group and −20 ml for the OE-group. Thus, the patients were on average close to “normovolemic” by the end of surgery supported by stable hemodynamic variables during the thoracic procedure and increasing plasma proANP (Table 3). Yet, from determination of plasma proANP, the patients seemed functional hypovolemic during the abdominal part of the procedure and more so in the RE-group that was exposed to head-up tilt combined with pneumoperitoneum. Thus, plasma proANP normalized when the RE patients were prepared for thoracic surgery, although plasma proANP did not reach the baseline value. In a similar study, plasma proANP was followed during open (ORC) and robot-assisted cystectomy (RARC) [13]. Plasma proANP decreased by 23% in the ORC-group with a 1500 ml positive fluid balance. Furthermore, plasma proANP correlated to fluid balance. Also, plasma proANP did not change for the RARC group, probably reflecting that the patients were head-down tilted and the blood loss was minimal.

As estimated by linear regression, a positive fluid balance by approximately 2400 (RE) and 2000 ml (OE) seemed to be required to keep plasma proANP stable during surgery (Fig. 2), i.e. approximately 600 ml more than was administered. Similarly for patients undergoing laparoscopic cholecystectomies in a head-up tilted position [1], improvement was found for pulmonary function, exercise capacity, nausea, and dizziness for patients administered 40 ml/kg LR (approx. 3000 ml) compared to 15 ml/kg (approx. 1000 ml). Yet, it remains to be determined whether more fluid should be administered during head-up tilted major abdominal procedures to prevent functional hypovolemia, i.e. whether it favors outcome including microcirculation to the anastomotic area and reduction in hypotensive episodes during surgery. On the other hand, excess fluid administration can lead to interstitial edema, impaired tissue healing with impact of anastomotic healing and also cardiopulmonary complications representing a risk factor [3, 24, 25].

Epidural anesthesia reduces SVR [26] due to sympatholysis as observed in the OE-group while SVR remained stable in the RE-group, besides a transitory increase may be due to CO_2_ insufflation with release of hormones like noradrenalin, compression of the splanchnic organs, and CO_2_ absorbed from the peritoneal cavity [27, 28]. Moreover, traction of the viscera can stimulate release of vasoactive hormones like prostacyclin corroborating a decrease in SVR, more prevalent during open than laparoscopic surgery [29–31].

This study was based on a secondary analysis of prospective data in a non-randomized design and selection bias should be considered. The two groups of patients demonstrated different baseline plasma proANP (Table 3) maybe related to that the ASA score was higher in the OE group and not acknowledged heart disease may have been overrepresented [32] supported by a higher prevalence of hypercholesterolemia. Thus, we cannot rule out that patients presenting co-morbidity have been selected for the “conservative” OE procedure although the primary criterion for selection of OE or RE was based on the access to the da Vinci System.

We accept that this report is only the second to evaluate fluid balance during surgery in relation to plasma proANP and that a wider database is needed to guide inter-operative fluid therapy on its influence on plasma proANP. However, we find it of interest to add a biomarker to evaluation of perioperative fluid balance presently mainly based on a predefined fluid regime supplemented by recording of physiological variables. Furthermore, we acknowledge that other measures of CO like transesophageal echocardiography [33], thoracic electrical impedance [34], and central venous oxygen saturation [35] can be obtained for assessment of CBV and may be required to generalize the findings of this study.

## Conclusion

This study showed a marked decrease in plasma proANP during the abdominal part of esophagectomy supporting that CBV is compromised during surgery and especially so for laparoscopic procedures including head-up tilt. We demonstrated a correlation between plasma proANP and perioperative fluid balance based mainly on LR. Taking plasma proANP to indicate filling of the heart, the data support that plasma proANP is a marker of fluid balance during surgery. Based on that idea, a fluid surplus by about 2200 ml would be required to maintain plasma proANP during esophagectomy. Yet, it needs to be evaluated whether a perioperative fluid regime directed to maintain plasma proANP improves outcome after surgery.

## Abbreviations

ANP: Atrial natriuretic peptide
ASA: American Society of Anesthesiologists classification
CBV: Central blood volume
CO: Cardiac output
HR: Heart rate
IR: Interquartile range
LR: Lactated Ringer’s
MAP: Mean arterial pressure
OE: Open esophagectomy
ORC: Open cystectomy
proANP: Pro-atrial natriuretic peptide
RARC: Robot-assisted cystectomy
RE: Robot-assisted esophagectomy
SV: Stroke volume
SVR: Systemic vascular resistance
